# Study on Absorption Mechanism and Tissue Distribution of Fucoidan

**DOI:** 10.3390/molecules25051087

**Published:** 2020-02-28

**Authors:** Xu Bai, E Zhang, Bo Hu, Hao Liang, Shuliang Song, Aiguo Ji

**Affiliations:** 1 Marine College, Shandong University, Weihai 264209, China; 15634407267@163.com (X.B.); Ezhang815@126.com (E.Z.); hobophar@163.com (B.H.); lianghao@sdu.edu.cn (H.L.); 2 School of Pharmaceutical Sciences, Shandong University, Jinan 250012, China

**Keywords:** fucoidan, fluorescent labeling, clathrin, absorption, tissue distribution.

## Abstract

Fucoidan exhibits several pharmacological activities and is characterized by high safety and the absence of toxic side effects. However, the absorption of fucoidan is not well-characterized. In the present study, fucoidan were labeled with fluorescein isothiocyanate (FITC) and their ability to traverse a monolayer of Caco-2 cells was examined. The apparent permeability coefficients (Papp × 10^−6^) of FITC-labeled fucoidan (FITC-fucoidan) were 26.23, 20.15, 17.93, 16.11 cm/sec, respectively, at the concentration of 10 μg/mL at 0.5, 1, 1.5 and 2 h. The absorption of FITC-fucoidan was suppressed by inhibitors of clathrin-mediated endocytosis, chlorpromazine, NH_4_Cl, and Dynasore; the inhibition rates were 84.24%, 74.61%, and 63.94%, respectively. This finding suggested that clathrin-mediated endocytosis was involved in fucoidan transport. Finally, tissue distribution of FITC-fucoidan was studied in vivo after injection of 50 mg/kg body weight into the tail vein of mice. The results showed that FITC-fucoidan targeted kidney and liver, reaching concentrations of 1092.31 and 284.27 μg/g respectively after 0.5 h. In summary, the present work identified the mechanism of absorption of fucoidan and documented its tissue distribution, providing a theoretical basis for the future development of fucoidan applications.

## 1. Introduction

**Figure 1:**
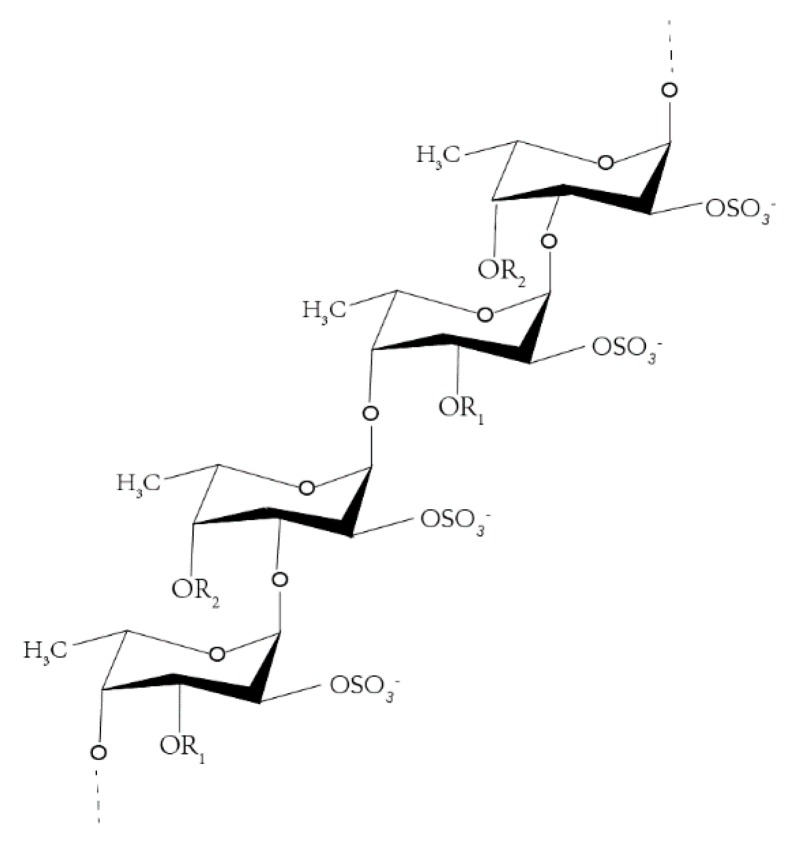
Fucoidan structure from *Fucus vesiculosus*.

Fucoidan is a water-soluble heteropolysaccharide, derived mostly from brown algae, such as *Fucus vesiculosus* (Figure 1) [1] and certain echinoderms [2,3]. The structure of fucoidan varies among species, whose skeleton mostly contains sulfate substituents and pyranose or other glycosyl unit, but the main structural unit consists of sulfated L-fucose [4]. As a naturally occurring chemical, the distribution of its relative molecular mass ranges from 1 to 1000 kDa [5]. The SO4^2−^ is the main functional group responsible for the biological properties of polysaccharides, and its quantity and position are critical determinants of the activity of these macromolecules. Recent studies have shown that fucoidan can exert a wide range of pharmacological effects, including anti-inflammatory [6], antitumor [7], antioxidative [8], antiviral, and antithrombotic activity, as well as improving immune response and lipid metabolism [5,9,10,11,12]. However, only a small number of studies addressed the mechanism of absorption and tissue distribution of this compound in vivo given their high molecular size [13,14,15,16]. Therefore, a detailed knowledge of its absorption mechanism is important for its biological activities.

Given the complex chemical structure of fucoidan, the difficulty of the mechanism of absorption determination has impeded the development of research on intestinal absorption of this molecule. Recently, techniques based on the detection of fluorescence have been employed in drug microanalysis because of their specificity, sensitivity, and low detection threshold [17]. Although fucoidan lacks chromogenic groups, fluorescent reagents can bind to the hemiacetal aldehyde group at the end of the polysaccharide molecule and generate a fluorescent moiety capable of absorbing ultraviolet light under certain conditions. The objective of the current study was to optimize the method of fluorescent labeling of fucoidan by fluorescein isothiocyanate (FITC) [18] and elucidate the mechanism of fucoidan absorption utilizing the Caco-2 cell 7-day model.

Although it is known that the tissue distribution of polysaccharides depends largely on their type and physicochemical properties [19,20,21], studies on this subject are limited. Therefore, the understanding of the mechanism of absorption and pharmacokinetics of fucoidan will not only promote the high-value development of kelp resources but will also provide the theoretical foundation for the application of fucoidan in healthcare products and pharmaceuticals.

## 2. Results

### 2.1. Fluorescence Labeling of Fucoidan

**Figure 2:**
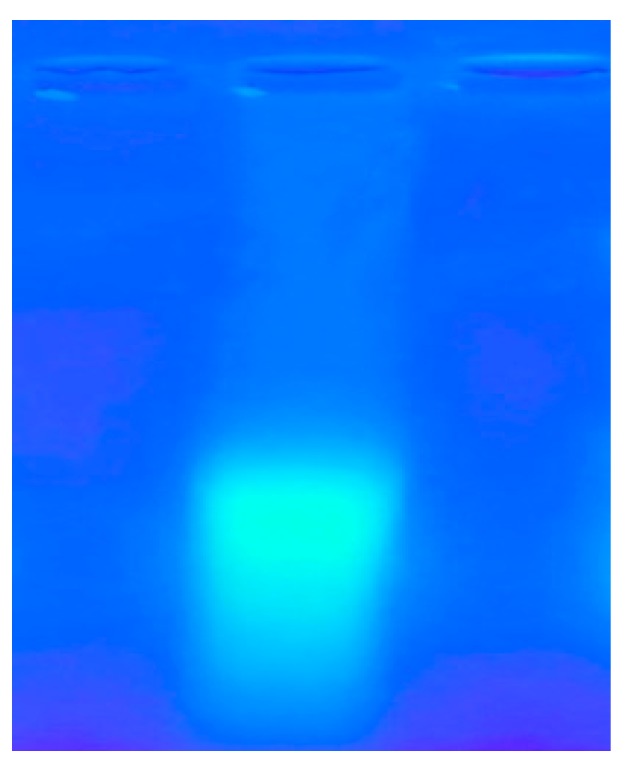
Agarose electrophoresis of fluorescein isothiocyanate-fucoidan (FITC-fucoidan).

The results of the photography under the gel imager showed that FITC-fucoidan had been marked successfully (Figure 2). Fucoidan had been successfully labeled. Specifically, the fucose content of fucoidan and FITC-fucoidan was 42.86% and 45.11%, respectively (Table 1), indicating that the method of FITC labeling did not affect the fucose content in fucoidan. 

### 2.2. Establishment and Assessment of Seven-Day Absorption Model of Caco-2 Cells

**Figure 3:**
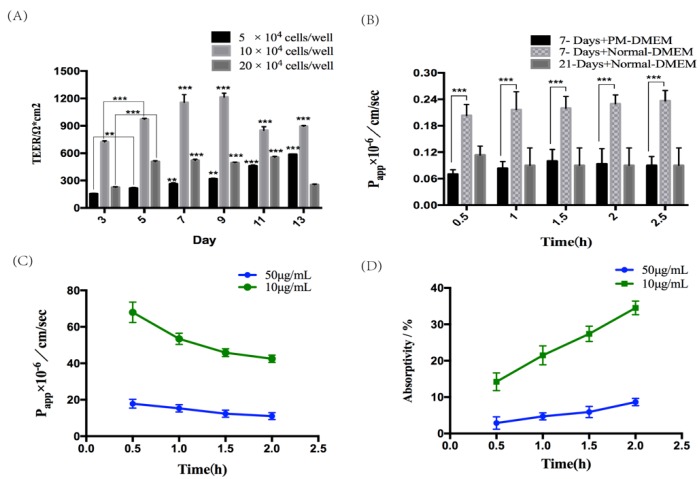
Establishment and assessment of seven-day absorption model of Caco-2 cells. (**A**) The transmembrane resistance (TEER) comparison at different concentration of Caco-2 cell density which were cultured in Puromycin -Dulbecco’s modified Eagle’s medium (PM-DMEM) medium. (**B**) The P_app_ of the markers of paracellular transport was expressed as the means ± SD (*n* = 3). (**C**) The Papp of FITC-transferrin at different time and concentration was expressed as the means ± SD (*n* = 3). (**D**) The absorptivity of FITC-transferrin at different time and concentration was expressed as the means ± SD (*n* = 3).

The transmembrane resistance (TEER) values of the monolayer of Caco-2 cells at different plating densities were tested (Figure 3A), showing that either too high or too low cell density was not conducive to the formation of the cell monolayer. At the appropriate cell density, the formation of monolayers can be accelerated in a short time by adding puromycin (PM). Therefore, Caco-2 cells were cultured in Puromycin -Dulbecco’s modified Eagle’s medium (PM-DMEM) at a density of 10 × 10^4^ cells/well for 7 days [22]. Together, the monolayers of Caco-2 cells cultured in PM-DMEM medium were compact and intact for 7 days, which had no significant difference from the 21-day model and could be used for drug absorption study (Figure 3B).

### 2.3. Verification of the Absorption and Transport Function of Caco-2 Monolayer Cell Model 

FITC-Transferrin is often used to test the function of Caco-2 monolayer cell model and it was transported from upper chamber to the lower chamber, which can be concluded that the 7-day absorption model of Caco-2 cells had been successfully established and exhibited adequate absorption and transport characteristics. At different concentration and time, the Papp and absorptivity of FITC-Transferrin with 10 μg /mL were higher than those of FITC-Transferrin with 50 μg /mL, and the lower the concentration, the easier it was to be absorbed (Figure 3C,D). So, it was speculated that the absorption and transport of transferrin was saturated.

### 2.4. The Mechanism of Fucoidan Absorption and Transport

#### 2.4.1. Absorption and Transport of Fucoidan

**Figure 4:**
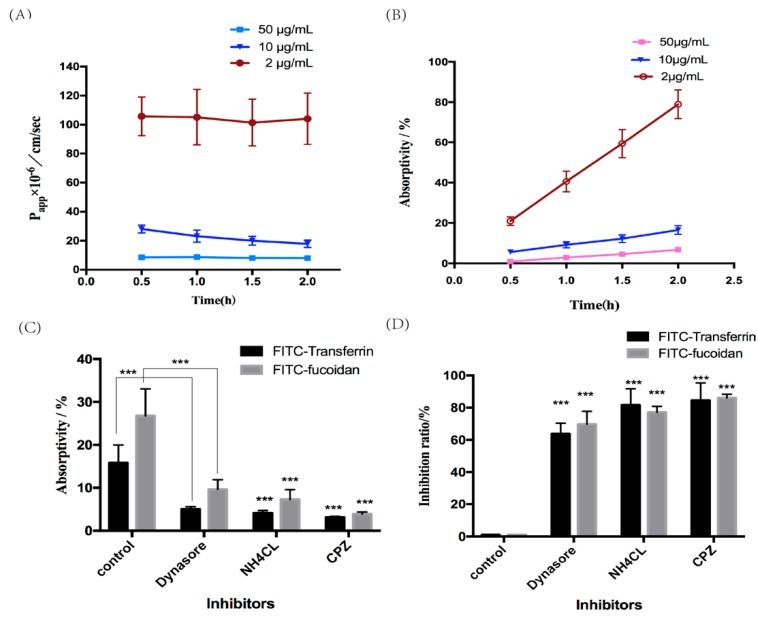
The absorption of FITC-fucoidan and the effect of inhibitors on it. (**A**) The Papp of FITC-fucoidan at different time and concentration was expressed as the means ± SD (*n* = 3). (**B**) The absorptivity of FITC-fucoidan at different time and concentration was expressed as the means ± SD (*n* = 3). (**C**) The absorptivity of FITC-transferrin and FITC-fucoidan by adding clathrin inhibitors CPZ, Dynasore and NH_4_CL was expressed as the means ± SD (*n* = 3). (**D**) The inhibition rate of FITC-Transferrin and FITC-fucoidan by adding clathrin inhibitors CPZ, Dynasore and NH_4_CL was expressed as the means ± SD (*n* = 3).

FITC-fucoidan did not affect the proliferation of the cells at concentrations of up to 1000 μg/mL, indicating the absence of a toxic effect. The Papp and absorption rates of FITC-fucoidan showed a trend consistent with the values obtained for FITC-transferrin, they decreased with increasing concentration (Figure 4A,B). These findings suggested that the transport of fucoidan may be carrier-dependent since transferrin is often used as a marker for clathrin-mediated endocytosis [23,24]. 

#### 2.4.2. Effect of Inhibition of Clathrin-Mediated Endocytosis on the Absorption and Transport of Fucoidan

Similar to FITC-Transferrin, Chlorpromazine (CPZ), Dynasore and NH_4_CL can inhibit FITC-fucoidan absorption. Compared with the control group, Papp values of Dynasore group, NH_4_CL group and CPZ group were 8.07, 5.68 and 3.53 cm/sec respectively, the absorption rate were 4.88%, 2.08%, and 2.13%, respectively (Figure 4C,D). CPZ, Dynasore and NH_4_CL are inhibitors of clathrin, thus, inhibitors of clathrin-mediated endocytosis reduced the absorption of FITC-fucoidan, demonstrating the involvement of the clathrin endocytic pathway in the absorption and transport of fucoidan.

### 2.5. Tissue Distribution of Fucoidan in Mice

#### 2.5.1. Toxicity of Fucoidan in Mice

During the observation period, the mice’s eating, drinking and excretion activities were normal, and the mice’s weight gain had no significant difference. The doses of FITC-fucoidan ranging from 10 to 2500 mg/kg body weight did not cause any mortality, and all mice survived the observation period, indicating the safety of FITC-fucoidan. Post-mortem examination showed that the morphology and color of all organs were normal, and no other pathological changes were found, providing additional documentation of FITC-fucoidan safety.

#### 2.5.2. Tissue Distribution of Fucoidan

**Figure 5:**
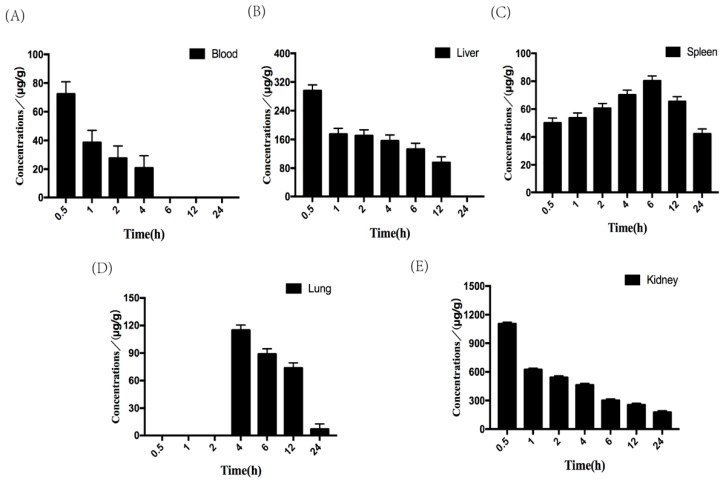
Tissue distribution of FITC-fucoidan in mice. (**A**) Concentrations of FITC-fucoidan in blood (μg/g) was expressed as the means ± SD (*n* = 5). (**B**) Concentrations of FITC-fucoidan in liver (μg/g) was expressed as the means ± SD (*n* = 5). (**C**) Concentrations of FITC-fucoidan in spleen (μg/g) was expressed as the means ± SD (*n* = 5). (**D**) Concentrations of FITC-fucoidan in lung (μg/g) was expressed as the means ± SD (*n* = 5). (**E**) Concentrations of FITC-fucoidan in kidney (μg/g) was expressed as the means ± SD (*n* = 5).

It has been confirmed that free FITC did not affect the detection of FITC-fucoidan in biological samples. The standard curve of FITC-fucoidan in various tissues was tested (Table 2). The concentrations of FITC-fucoidan in various tissue samples at different times (Figure 5A–E) and the main pharmacokinetic parameters of FITC-fucoidan (50 mg/kg) after tail vein injection in mice were tested (Table 3). These results documented that FITC-fucoidan was rapidly eliminated from the blood after intravenous administration; the blood concentration of FITC-fucoidan reached 66.37 μg/g after 30 min and decreased afterward; FITC-fucoidan was not detected in the blood after 4 h. The presence of FITC-fucoidan was identified in the liver, spleen, lung, and kidney, with the latter always exhibiting the highest concentration. The level of FITC-fucoidan in the kidney tissue reached 1092.31 μg/g after 4 h, followed by a decrease in concentration, indicating that the kidney has a strong ability to uptake FITC-fucoidan. The concentration of FITC-fucoidan in the liver reached a maximum of 284.27 μg/g at 0.5 h, and in the lung a maximum of 110.92 μg/g at 4 h. FITC-fucoidan was always detected in the spleen, and the concentration reached a maximum of 77.79 μg/g at 6 h. The molecule was not detected in the brain and the heart.

## 3. Materials and Methods 

### 3.1. Materials

Fucoidan from *F. vesiculosus* was purchased from Sigma-Aldrich, USA (≥95%, CAS:9072-19-9), its monosaccharide composition has been detected (fucose:138.7 ± 5.5; rhamnose: 2.0 ± 0.6; galactose: 27.9 ± 1.4; glucose: 2.5 ± 1.8; xylose: 12.8 ± 1, 6; mannose: 0.2 ± 0.4: glucuronic acid: 18.5 ± 1.9) [25,26]; Caco-2 cell was purchased from Kunming Institute of Zoology, Chinese Academy of Sciences Kunming Cell Bank; Trypsin was purchased from Invitrogen, US; Penicillin and Streptomycin was purchased from Invitrogen, USA; Chromatographic column (G4000PWXL) was purchased from Agilent, USA; Methylthiazolyldiphenyl-tetrazolium bromide (MTT) was purchased from Beyotime Biotechnology, China; DMSO was purchased from Sigma-Aldrich, USA; puromycin was purchased from Aladdin, China; fluorescent yellow was purchased from Aladdin, China; chlorpromazine hydrochloride was purchased from Aladdin, China; Dynasore was purchased from Aladdin, China; Ammonium chloride was purchased from Shanghai Sinopharm Group, China; FITC labeled human transferrin (Chromatographic grade) was purchased from Jackson, USA; Fluorescein isothiocyanate (FITC) was purchased from Aladdin, China; Sodium cyanoborohydride was purchased from Aladdin, China; Fucose was purchased from L-Fucose, Aladdin, China; Transwell Chamber (0.4 μm) was purchased from Millipore, USA.

### 3.2. Fluorescent Labeling of Fucoidan

After dissolving 1 g of fucoidan in phosphate buffer solution (pH 7.4), tyramine (2g) was added and the reaction was carried out at 37 °C for 24 h. Subsequently, NaBH_3_CN (1g) was added, the sample was placed in a shaker at 37 °C for 96 h, and then centrifuged to obtain a supernatant. The FITC-labeled fucoidan (FITC-fucoidan) solution was obtained by reacting the supernatant with 25 mg FITC at 37 °C for 36 h. The resulting solution was centrifuged, and the supernatant was subjected to ultrafiltration using a tangential flow membrane filtration system (Spectrum Laboratories, Inc, USA). Small molecules were removed by Superdex 30 (Thermo Fisher Scientific) and the preparation was freeze-dried to obtain pure FITC-fucoidan [25]. 

### 3.3. Effect of FITC Labeling on the Content of Fucose and Sulfate

Using fucoidan and FITC-fucoidan as samples to be tested, the changes of fucose content and sulfate content in fucoidan were determined by cysteine hydrochloride method and barium chloride-gelatin method respectively [27,28].

### 3.4. MTT Assay for Cell Viability 

The cytotoxicity of FITC-fucoidan to Caco-2 cells was determined using the MTT assay. Precultured cells (5 × 10^4^ cells/well) in DMEM medium were plated on a 96-well microplate for 24 h. Subsequently, the medium was removed and replaced with medium containing different concentrations of FITC-fucoidan (0, 10, 100, 1000 μg/mL). After incubation for 48 h, 10 μL of 5 mg/mL MTT solution was added to each well, and the cells were incubated for an additional 4 h. The medium was aspirated, 100 μL of DMSO was added to each well, and the plates were shaken for 3 min using a vortex instrument until thoroughly mixed. The absorbance of each well was measured at a wavelength of 490 nm by a microplate reader [28].

### 3.5. Caco-2 Cell Culture and Establishment of Caco-2 Monolayer Cell Model

Caco-2 cells were cultured in DMEM containing 20% (*v/v*) FBS, penicillin and streptomycin (100 U/mL), and the cells were cultured in a 25 cm^2^ cassette culture flask and placed in a CO_2_ incubator (Thermo Electron Corporation, USA). When the cell density reached 80–90% confluence, it was digested with 0.25% trypsin-0.02% Ethylene Diamine Tetraacetic Acid (EDTA) and passaged at a ratio of 1:3. Cells in the logarithmic phase of growth were suspended at concentrations of 25 × 10^4^, 50 × 10^4^, and 100 × 10^4^ cells/mL in DMEM containing 0.4 μg/mL puromycin (PM). The cells in PM-DMEM were cultured for 7 days, and the cells in normal-DMEM for 21 days. Add 1.0 mL of medium to the lower chamber of Transwell. The liquid was changed every other day [29]. 

After the cells were cultured for 21 days in normal-DMEM and 7 days in PM-DMEM, a Caco-2 cell monolayer model was formed by using a Millicell-ERS voltammeter (Millipore Company, USA) to monitor the transmembrane resistance (TEER) of the Caco-2 cell monolayer film in real time to evaluate the quality of the monolayer cell membrane. The TEER value of the Caco-2 cell monolayers exceeding >500 Ω × cm^2^ was considered to be qualified for subsequent transmembrane transport experiments. After modeling, 250 μg/mL fluorescent yellow solution was added to the apical side to detect the transmittance of basal side fluorescent yellow at different times. The fluorescence intensity of the medium was measured by a microplate reader at 490 nm and at 520 nm.

### 3.6. Verification of the Absorption and Transport Function of Caco-2 Monolayer Cell Model

After a seven-day absorption model of Caco-2 cells was established, 300 μL of FITC-labeled transferrin (FITC-transferrin) solution (10 or 50 μg/mL) was added to the upper chamber, and 800 μL of DMEM medium was added to the lower chamber. The chambers were incubated at 37 °C, and the medium from the lower chamber was collected at 30, 60, 90, 120, and 150 min. The fluorescence intensity of the medium was measured by a microplate reader at 490 nm and at 520 nm [29]. 

### 3.7. The Mechanism of Fucoidan Absorption and Transport

After establishing a 7-day absorption model of Caco-2 cells in a transwell chamber, 300 μL of FITC-fucoidan solution, at concentrations of 2, 10, and 50 μg/mL, was added to the upper chamber, and 800 μL of DMEM medium was added to the lower chamber. The chambers were incubated at 37 °C, and the medium from the lower chamber was collected at 30, 60, 90, 120, and 150 min. The intensity of fluorescence was measured by a microplate reader at 490 nm and 520 nm. 

The cell monolayers were incubated with solution containing different inhibitors (CPZ, Dynasore, and NH_4_Cl in DMEM medium at concentrations of 10, 20, and 50 μg/mL). The experiments included two groups, the first group is as follows: blank group, FITC-fucoidan group, CPZ + FITC-fucoidan group, NH_4_Cl + FITC-fucoidan group, Dynasore+FITC-fucoidan group; the second group is as follows: FITC-transferrin group, CPZ + FITC-transferrin group, NH_4_Cl + FITC-transferrin group, Dynasore+FITC-transferrin group. Inhibitors were added to the upper chamber respectively, and DMEM medium was added to the lower chamber. After 30 min, the medium in the upper chamber was aspirated, and 10 μg/mL of FITC-fucoidan or FITC-transferrin solutions were added to the upper chamber. DMEM medium was added to the lower chamber and the culture was incubated at 37 °C for 120 min. Finally the medium from the lower chamber was collected at 30, 60, and 120 min. The intensity of fluorescence was measured by a microplate reader at 490 nm and 520 nm. 

### 3.8. Tissue distribution of Fucoidan in Mice

Twenty Kunming mice were kept under a 12-h light–dark cycle at 22 ± 2 °C and humidity of 60 ± 10%; water and food were available ad libitum. Mice were fasted overnight before the experiment. The mice were weighed and randomly divided into 4 groups, the blank group was injected with normal saline, and the three remaining groups were injected into the tail vein with 10, 500, and 2500 mg/kg FITC-fucoidan for 7 days, respectively. The weight was measured again at the end of the experiment to determine the toxicity of fucoidan on mice. 

The untreated mice were sacrificed, a standard curve was established as the following method. Blood was quickly collected from the heart, centrifuged in a tube containing 0.2 mL of 4% trisodium citrate, and the supernatant plasma was aspirated and frozen. Heart, liver, spleen, lung, kidney, and brain tissue were collected and homogenized. Tissue homogenate and plasma were added with 0, 0.2, 0.4, 0.8, 1, 2, 4, 6, 8, 10, 15, 20, 25, 30, 35, 40, 45, 50, 60, 80 and 100 μL of FITC solutions respectively to obtain FITC-tissue homogenate mixture. The fluorescence intensity was measured at 490 nm and 520 nm using a microplate reader.

The treated mice was injected into the tail vein with a solution of FITC-fucoidanl (50 mg/kg). The blank group received an injection of normal saline. Mice were sacrificed at 0.5, 1, 2, 4, 6, 12, and 24 h after administration of FITC-fucoidan. All tissues and organs were collected and homogenized according to the same method as above. The intensity of fluorescence was measured at 490 nm and 520 nm using a microplate reader. PK Solver 2.0 and non-atrioventricular model were used to analyze the pharmacokinetic parameters of the experiment [30,31].

### 3.9. Statistical Analysis

All data in graphs were presented as the mean value ± standard deviation from three independent measurements. The statistical analysis was used in statistical software (SPSS 21.0.0.0, Chicago, Ill, USA) and GraphPad Prism 7.00 (GraphPad Software, California, USA). *P* < 0.05 was considered significant.

## 4. Conclusions

In recent years, much has been learned about the biological activities of fucoidan. However, previous studies on the mechanism of polysaccharide transport in the intestine have focused on oligosaccharide and high molecular weight polysaccharides was less successful. The molecular mechanisms that allow high molecular weight polysaccharides to cross the intestinal epithelial cell monolayers are critical for its clinical application. In this study, we found that absorption of fucoidan was negatively correlated with its concentration before reaching dynamic equilibrium. It is speculated that the absorption and transport of fucoidan need carriers and the endocytosis pathway of reticulin is involved in their absorption. With the further development of the experiment, the tissue distribution of fucoidan in mice was explored. The experimental results showed that fucoidan rapidly distributed from blood to tissues, and accumulated preferentially to kidney and liver, but it was not detected in the heart and brain tissues in this experiment, which can provide a theoretical basis for the future development of fucoidan applications.

In order to explain the absorption mechanism of fucoidan more clearly, more in-depth studies on gene and protein levels are needed in the future to lay a deeper theoretical foundation for the development of fucoidan.

## Figures and Tables

**Table 1:** The fucose content and sulfate content of fucoidan before and after being labeled.

Fucoidan	Content%	
	Before Labeled	After Labeled
Fucose content	42.86 ± 0.24	45.11 ± 0.88
Sulfate content	25.37 ± 0.25	25.33 ± 0.34

**Table 2:** The standard curve of FITC-fucoidan in tissues.

Tissue	Standard Curve	R^2^
Blood	Y = 1028.62 X + 48217.63	0.999
Heart	Y = 1595.96 X + 153165.43	0.994
Liver	Y = 3217.36 X + 164583.03	0.998
Spleen	Y = 2902.54 X + 26586.43	0.998
Pulmonary	Y = 3194.69 X + 35547.02	0.995
Renal	Y = 1791.22 X + 215822.65	0.99
Brain	Y = 2427.88 X + 48044.49	0.998

**Table 3:** Main pharmacokinetic parameters of FITC-fucoidan in mice tissues by intravenous administration (50 mg/kg).

Parameter	Unit	Tissue				
		Blood	Liver	Spleen	Lung	Kidney
K_el_	1/h	0.25 ± 0.10	0.07 ± 0.01	0.003 ± 0.02	0.18 ± 0.05	0.03 ± 0.04
T_1/2_	h	2.77 ± 0.82	10.67 ± 3.73	209.22 ± 6.94	3.79 ± 0.97	22.27 ± 1.75
T_max_	h	0.5	0.5 ± 0.29	6 ± 1.15	4 ± 1	0.5
C_max_	μg/g	66.37 ± 25.56	284.27 ± 211.88	77.79 ± 30.05	110.92 ± 81.897	1092.31 ± 297.66
C_0_	μg/g	135.28 ± 59.91	495.25 ± 159.14	47.61 ± 17.28	188.58 ± 48.32	1949.94 ± 1448.45
AUC _0-t_	μg/g×h	138.71 ± 20.64	1653.86 ± 567.04	1597.28 ± 394.38	1694.21 ± 580.70	7520.11 ± 2110.44
AUC _0-∞_	μg/g×h	198.11 ± 41.10	2947.506 ± 992.52	22886.97 ± 1301.13	1709.85 ± 588.22	12834.30 ± 5247.13
MRT _0-∞_	h	3.23 ± 1.30	14.66 ± 5.94	303.96 ± 13.44	6.88 ± 0.37	28.14 ± 12.324
CL	(mg)/(μg/g)/h	0.25 ± 0.04	0.02 ± 0.001	0.002 ± 0.01	0.03 ± 0.004	0.004 ± 0.003
